# In vitro transdifferentiation of human adipose tissue-derived stem cells to neural lineage cells - a stage-specific incidence

**DOI:** 10.1080/21623945.2019.1607424

**Published:** 2019-04-29

**Authors:** Subathra Radhakrishnan, Omana Anna Trentz, Mettu Srinivas Reddy, Mohamed Rela, Mahesh Kandasamy, Shanmugaapriya Sellathamby

**Affiliations:** a National Foundation for Liver Research (NFLR), Gleneagles Global Health City, Chennai, India; b Department of Biomedical Science, Bharathidasan University, Tiruchirappalli, India; c MIOT Institute of Research (MIR), MIOT International, Chennai, India; d Institute of Liver Disease and Transplantation, Gleneagles Global Health City, Chennai, India; e Department of Animal Science, Bharathidasan University, Tiruchirappalli, India

**Keywords:** IFP, ADSC, NPC, Woodbury’s method, chemical induction, glial progenitors

## Abstract

The present Study investigated the intrinsic ability of adipose tissue-derived stem cells (ADSCs) and their neural transdifferentiation in a stage-specific manner. Woodbury’s Chemical induction was implemented with modifications to achieve neural transdifferentiation. In Group I, ADSCs were preinduced with β-mercaptoethanol (β-ME) and later, with neural induction medium (NIM). In Group II, ADSCs were directly treated with NIM. In Group III, a DNA methyltransferase (DNMT) inhibitor 5-azacytidine was applied to understand whether transdifferentiation is controlled by epigenetic marks. Irrespective of the presence of (β-ME), the differentiation protocol resulted in glial-lineage cells. Group III produced poorly -differentiated neural cells with neuron-specific enolase positivity. A neuroprogenitor stage (NPC) was identified at d 11 after induction only in Group I. In other groups, this stage was not morphologically distinct. We explored the stage-specific incidence NPC, by alternatively treating them with basic fibroblast growth factor (bFGF), and antioxidants to validate if different signalling could cause varied outcomes (Group IV). They differentiated into neurons, as defined by cell polarity and expression of specific proteins. Meanwhile, neuroprogenitors exposed to NIM (Group I) produced glial-lineage cells. Further refinement and study of the occurrence and terminal differentiation of neuroprogenitors would identify a promising source for neural tissue replacement.

## 1. Introduction

Adipose tissue-derived stem cells (ADSCs) are envisioned to be the most suitable adult stem cells for regenerative therapy, particularly for treating neurodegenerative disorders, due to their easy availability and plasticity [1]. ADSCs represent a potential source of multipotent stem cells that can easily be obtained through minimally invasive procedures. For instance, the infrapatellar fat pad (IFP) resected as medical waste during total knee arthroplasty serves as a good source of ADSCs that are known to have good proliferative index [2]. ADSCs have the additional advantage of being capable of experimental differentiation into selected subpopulations of specific somatic cell types, including neurons. A number of diverse protocols has been implemented by various research groups [3,4] for the transdifferentiation of ADSCs to neurons. The experimental outcomes of these protocols were marginally described by neuronal morphology and neuronal cell type markers; however, an efficient, stage-specific protocol for the differentiation of ADSCs towards neural phenotype has not been well described to date. Furthermore, while substantial experimental information has been generated on the *in vitro* neuronal differentiation of bone marrow-derived mesenchymal stem cells (MSCs), very few studies have been conducted on the IFP–derived ADSCs [5]. Our team has previously reported the presence of embryonic stem cell markers in infrapatellar fat pad-derived stem cells, as well as the inherent differentiation potential of ADSCs towards neuronal lineage cells without any specific induction [2,6]. However, there remains no method for signal-induced neuronal differentiation of IFP derived – ADSCs with biomimetic stage-specific markers.

Previously, Woodbury et al. [7,8] demonstrated the transdifferentiation ability of human bone marrow-derived MSCs to neurons upon β-mercaptoethanol treatment [7,8]. As MSCs share many biological features with IFP-derived ADSCs, Woodbury’s protocol has been adapted for the neural differentiation of ADSCs with minor modifications. This well-known method was experimentally revisited as the stage-specific incidence of ADSC differentiation needs to be validated.

The main objective of the current study was to investigate how the IFP-derived ADSCs acquire neuronal phenotype in a sequential manner while undergoing differentiation. To further confirm the fidelity of transdifferentiation and to determine if the outcome is a result of morphological artefacts, cell fusion or gene transfer, or the presence of contaminating cells, we examined a specific neuronal stage with a new array of neuronal-specific biomarkers. It has been suggested that true differentiation could possibly be achieved only by the modulation of epigenetic parameters [9,10]; we therefore also examined whether the transdifferentiation of ADSCs is controlled by DNA methyltransferases (DNMTs). These are highly conserved proteins responsible for both *de novo* and maintenance methylation in the genome, which epigenetically regulates gene expression. During the differentiation of pluripotent stem cells, pluripotent genes have been found to be silenced through increased DNA methylation at promoter CpG islands, mediated by DNMT, making the promoter region inaccessible to transcription machinery [11].

## 2. Materials and methods

### 2.1. Tissue collection and isolation of ADSCs

Written informed consent was obtained from patients after the provision of a detailed description of the experiment and prior to enrolment into the study. All procedures were carried out in accordance with the requirements of the Institutional Ethical Committee (IEC) and Institutional Committee for Stem Cell Research (ICSCR) of MIOT institute of Research and National Foundation for Liver research.

ADSCs were isolated from the IFP tissues obtained from 10 patients undergoing knee arthroplasty. All patients were between 60 and 72 y of age. The obtained fat was immediately transported to the laboratory in a sterile container containing normal saline, washed with sterile PBS, and digested with 0.075% collagenase I (PAN biotech). The digest was then filtered using a 70 μm cell strainer (BD Biosciences) and centrifuged to obtain the stromal vascular fraction in the pellet. The pellet was resuspended in culture medium and then plated. The isolation and characterization of ADSCs were performed as described previously [2,12].

### 2.2. Culture of ADSCs

ADSCs were cultured and maintained in Dulbecco’s-modified Eagle’s medium (DMEM) with 10% foetal bovine serum (FBS) and 60 µg of antibiotic-antimycotic (Invitrogen). Cultures were maintained in a 5% CO_2_ incubator with 97% humidity at 37^ο^C. Once the cells reached 80% confluency, they were subcultured. The cells used in the study were from passage two and three.

### 2.3. Neural induction in ADSC

Woodbury’s chemical induction method [7,8] was adopted for neural differentiation with few modifications. Specifically, the whole experiment was carried out in a complete medium (with 10% FBS), rather than serum-free medium, to provide attachment factors present in serum and to facilitate mechanical strength.

ADSCs were pre-incubated with complete medium containing 5 mM β-mercaptoethanol (β-ME) (pre-induction medium) for 24 h before subjecting the ADSCs to neural induction medium (NIM) containing 2% dimethyl sulfoxide (DMSO), 200 µM butylated hydroxyanisole (BHA), and 40 ng/ml basic fibroblast growth factor (bFGF). The cells were replenished with fresh NIM every 4 d and were passaged once they were 80% confluent. Cells were maintained in the same conditions for 45 d. (Group I – Modified Woodbury’s method) [7,8].

The second set of experiments was carried out without pre-induction. In this case, only NIM was added to the passage-three ADSCs; the pre-induction medium containing β-ME was not applied. The NIM was replaced every 4 d and cells were passaged once they were at 80% confluence. Cultures were maintained in the same medium for 45 d (Group II – Without Pre-induction).

#### 2.3.1 Neural induction in ADSCs with inhibitor of the epigenetic modulator DNMT, 5-azacytidine 

To understand whether the transdifferentiation of ADSCs is controlled by epigenetic modifications mediated by DNA methyltransferase (DNMT), 10 μM of the DNA methyltransferase inhibitor 5-azacytidine was used in both the preinduction and neural induction protocols (Group III – Neural Induction with 5-azacytidine).

### 2.4. Alternative treatment with bFGF and antioxidants in neuroprogenitors

To prove that the cells with small neurite projections at d 11 after induction (Group I) were neuroprogenitor cells (NPCs) and capable of differentiating into various cell types depending on the chemical and biological signals, they were subjected to an alternative treatment with bFGF and antioxidants (β-ME and BHA) rather than NIM. This assay is based upon terminal neuronal differentiation requiring the withdrawal of mitogens for at least one round after replication [13]. After pre-induction with β-ME and subsequent treatment with NIM (i.e. at d 11), the medium was removed, the cells were washed with PBS, and the culture was replenished with DMEM complete medium containing 50 ng/ml bFGF. After the 15th day, the medium was again removed and supplemented with DMEM complete medium containing 200 µM BHA (thereby achieving the withdrawal of bFGF/mitogens). After the 19th day, BHA was removed by washing with PBS, following which DMEM complete medium containing 50 ng/ml bFGF was added; the cells were maintained in this for 45 d (Group IV).

Thus, the NPCs resulted as a stage-specific incidence in group I undergone two different signals, NIM in Group I and Alternative treatment with bFGF and antioxidants in Group IV.

The different treatment groups in the neural induction protocol are clearly described in Table 1. ADSCs grown and passaged only in DMEM complete medium were used as a control in all experimental setups. After treatment, total cellular RNA was isolated and reverse transcriptase polymerase chain reaction (RT-PCR) used to evaluate the expression of neuronal-specific transcripts. In addition, immunocytochemistry (ICC) was used to evaluate cells for the expression of neuronal-specific proteins.

**Table 1.** Groups undergone neural induction, their respective controls and maintenance medium.

Group	Specification	Control	Pre-induction	Maintenance medium
I	Modified Woodbury method	ADSC	5mM β-ME	NIM
II	Modified Woodbury’s method without Preinduction	Group I	-	NIM
III	Modified Woodbury’s method with 5- Azacytidine (AZC)	Group I	10μM AZC **+** 5mM β-ME	NIM with 10μM AZC
IV	Neural induction in Neuroprogenitor-like cell	Group I	5mM β-ME	NIM (till d 11), later temporal bFGF treatment

### 2.5. Morphological analysis

Cell subjected to different treatment protocols were closely monitored for morphological alterations using an inverted phase contrast microscope (Nikon). Cells at different time points (d 11, 15, 25, and 45 after induction) showing characteristic morphological changes were evaluated for differential expression of neuronal-specific mRNA transcripts and proteins by semi-quantitative RT-PCR and western blot or immunofluorescence analysis, respectively. Cells were also cultured on sterile glass coverslips, fixed with 4% paraformaldehyde, and stained with Gill’s haematoxylin as per standard protocol. Finally, the cells were mounted using an aqueous mounting medium and observed under the microscope (Nikon Ts-100 F).

### 2.6. Immunocytochemistry

To validate whether cells with different morphologies at different days (d 11, 27, and 30 after induction) represented different neuronal stages, the expression of neuronal-specific proteins was evaluated using immunofluorescence staining. Cells were cultured on sterile glass coverslips, fixed with 4% paraformaldehyde (Sigma-Aldrich), and permeabilized with 0.2% Triton x-100 in PBS. To block non-specific sites, the cells were treated with blocking buffer containing 1% bovine serum albumin (BSA) in PBS for 1 h. Blocked cells were washed three times with washing buffer (PBS) and incubated overnight with the primary antibody at 4°C. The next day, the cells were washed extensively with PBS and incubated for 2 h with an appropriate secondary antibody. After incubation, the cells were washed three times with PBS, mounted with aqueous mounting medium, and visualized and photo-documented under a fluorescent microscope (Nikon Ts-100 F) at 494/520 nm. The stained cells were observed at the magnification of 20 X. All the antibodies used for immunocytochemical analysis were listed in Table 3.

**Table 2.** Primer sequences used for semi quantitative gene expression.

Gene	Primers	bp
GAPDH	For: 5ʹ-GGGCTGCTTTTAACTCTGCT-3ʹ Rev: 5ʹ-TGGCAGGTTTTTCTAGACGG-3’	702
Neuron Specific Enolase	For:5ʹ-CTGATGCTGGAGTTGGATGG-3ʹ Rev:5ʹ-CCATTGATCACGTTGAAGGC-3’	188
Neurofilament – L	For:5ʹ-TCCTACTACACCAGCCATGT-3ʹ Rev: 5ʹ-TCCCCAGCACCTTCAACTTT-3’	284
SNAP-25	For: 5ʹ- AGTTGGCTGATGAGTCGCTG-3 Rev:5ʹ-TGAAAAGGCCACAGCATTTC-3’	207
Claudin 11	For: 5‘-TGACCTGCGGCTACACCATCC-3‘ Rev: 5‘-AGGCACAGCACAGCACCAATCC-3‘	412
Midkine	For: 5‘-CGACTGCAAGTACAAGTTTGAGAAC-3‘ Rev: 5‘-TCTCCTGGCACTGAGCATTG-3‘	110
GFAP	For: 5ʹ-CCTCTCCCTGGCTCGAATG-3ʹRev:5ʹ-GGAAGCGAACCTTCTCGATGTA-3’	161
Nestin	For: 5ʹ-AGGATGTGGAGGTAGTGAGA-3ʹ Rev: 5ʹ-TGGAGATCTCAGTGGCTCTT-3’	251
ZNF521	For.: 5ʹ-GGTGAAACTTGATATCAATGGCC-3ʹ Rev.: 5ʹ-GGTGAAACTTGATATCAATGGCC-3’	482

**Table 3.** Antibodies used for immunocytochemistry and western blot.

S.No.	Primary /SecondaryAntibody	Company Provided Antibody	Catalogue number
1	CD 166	Santa Cruz	sc-74558
2	Nanog	Santa Cruz	sc-293121
3	Nucleostemin	Santa Cruz	sc-166460
4	CD105	Santa Cruz	sc-71042
5	CD13	Santa Cruz	sc-166270
6	NSE	Santa Cruz	sc-21738
7	NF-L	Santa Cruz	sc-71678
8	SNAP25	Santa Cruz	sc-7538
9	GAP 43	Santa Cruz	sc-7457
10	Olig2	Santa Cruz	sc-19967
11	Donkey anti-goat IgG-FITC	Santa Cruz	sc-2024
12	Mouse anti-goat IgG – FITC	Santa Cruz	sc-2356
13	m-IgGkBP-FITC	Santa Cruz	sc-516140
14	Goat anti mouse IgG - FITC	Santa Cruz	sc-2010
15	Mouse anti-goat IgG – HRP	Santa Cruz	sc-2354
16	m-IgGkBP-HRP	Santa Cruz	sc-516102

All the microscopic experiments were performed by obtaining samples from atleast three different patients, in triplicates.

### 2.7. RNA extraction and RT-PCR

Total RNA was extracted from 100% confluent cells using the RNeasy Mini kit (Qiagen). RNA was converted to complementary DNA (cDNA) using the Omniscript® Reverse Transcriptase kit (Qiagen) with a reaction volume of 20 µl at 37°C for 60 min. The cDNA was amplified via PCR using the HotStarTaq® Master Mix PCR kit (Qiagen) as recommended by the manufacturer.

#### 2.7.1 ADSC gene expression analysis

To analyse the expression of the zinc finger protein, ZNF521, total RNA was extracted from ADSCs on d 17, 32, 38 and 52 (The samples were obtained from atleast two patients and the experiment was performed in duplicates).

#### 2.7.2 Neural – lineage cells gene expression analysis

RNA was extracted from differentiated neural-lineage cells on day 25 after induction. Expression of the following genes was observed: neuron-specific enolase (NSE), neurofilament (NF-L), midkine (MDK) (also known as neurite growth-promoting factor 2 [NEGF2]), claudin 11 (CLDN11), and Glial fibrillary acidic protein (GFAP) (The samples were obtained from atleast two patients and the experiment was performed thrice).

The sequences of all the oligonucleotide primers are listed in Table 2. Glyceraldehyde-3-phosphate dehydrogenase (GAPDH) was used as an endogenous control.

### 2.8. Western blot analysis

Total protein was extracted from differentiated cells on d 25 after induction. ADSCs from d 20 served as control. The cells were washed three times with ice-cold PBS and lysed with 300 µl of ice-cold radio-immunoprecipitation assay (RIPA) buffer (Sigma-Aldrich) (with PMSF in ethanol). Total protein estimation was carried out by Lowry’s method. Subsequently, 30 μg of total cell lysate was loaded onto a 10% gel and separated by size through sodium dodecyl sulphate-polyacrylamide gel electrophoresis (SDS-PAGE). The resulting bands were transferred to a nitrocellulose membrane using a wet-blot transfer system with Tris-glycine buffer (50°C for 1 h at 100–200 milliamps). To block non-specific sites, the membrane was treated with 5% skimmed milk powder in PBS with 0.1% Tween-20 (PBST). The blocked membrane was washed and incubated overnight with GAP43-specific goat polyclonal primary antibody (Santa Cruz) (1:8000 dilution) at 4°C. The membranes were then incubated with donkey-derived anti-goat IgG-horseradish peroxidase (HRP) conjugated secondary antibody (1:1000) for 2 h at room temperature. After washing, the blots were developed using an enhanced chemiluminescence (ECL) kit (GE-Healthcare). Expression of Oligodendrocyte transcription factor 2 (OLIG2) was identified with OLIG2-specific mouse polyclonal primary antibody and appropriate secondary antibody from Santa Cruz. (The samples were obtained from two patients and the experiment was performed in duplicates)

## 3. Results

### 3.1. Characterization of ADSCs reveals their stemness and similarities to bone marrow-derived MSCs

**Figure 1.**
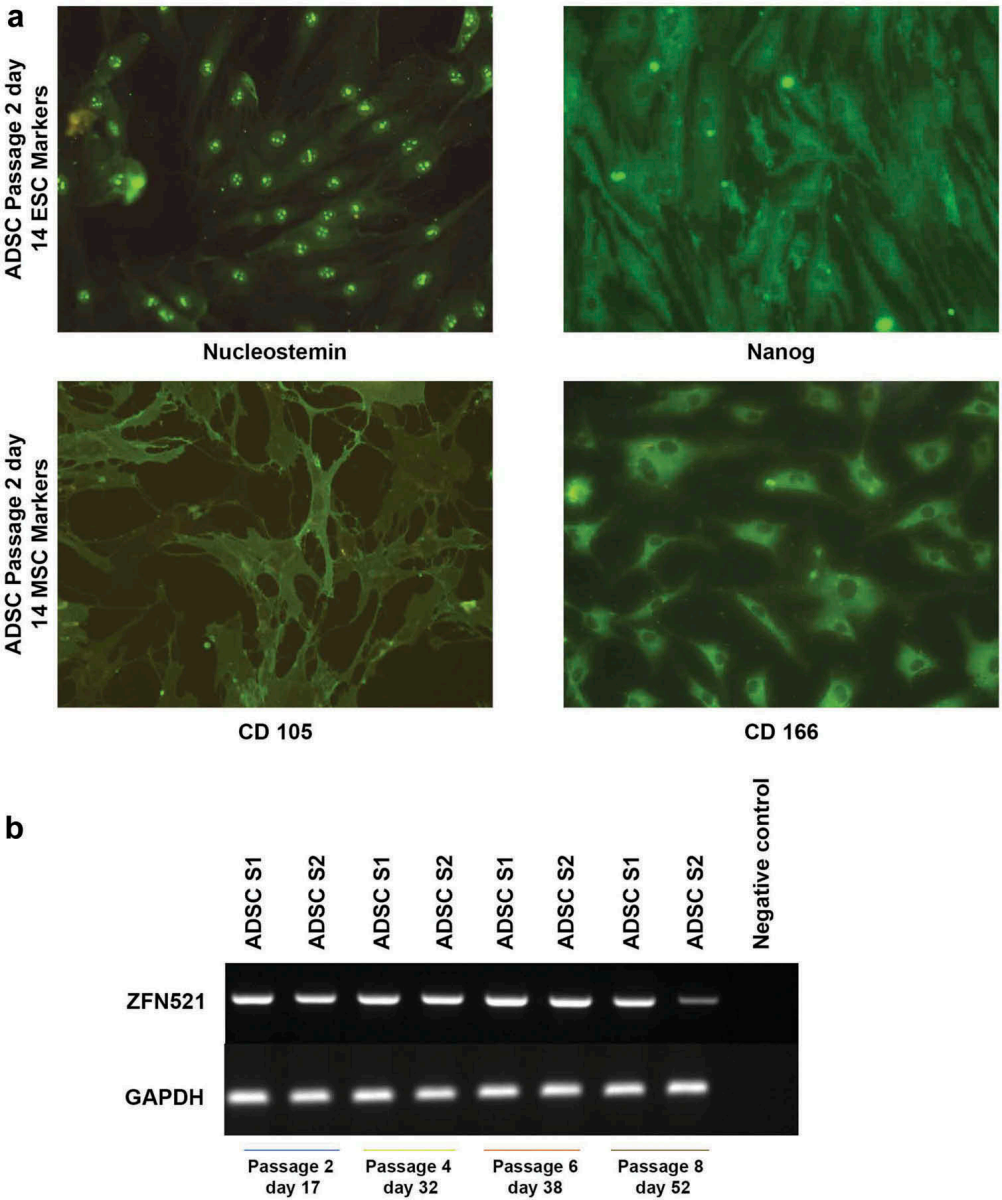
Immunofluorescence analysis displays the stemness of ADSCs (a). ADSCs exhibit embryonic stem cell marker (ESC) Nucleostemin, localized in the nucleolus, and Nanog homeobox, widely spread. Mesenchymal stem cell marker (MSC) CD105 is localized in the membrane, and CD166 is concentrated around the nucleus and diminished in the periphery (Magnification 20 x). Semi-quantitative gene expression analysis of the zinc finger protein *ZNF521* in ADSC samples 1 and 2 (ADSCS1 and ADSCS2) from d 17 to d 52 (b), with GAPDH as an endogenous control.

The expression of two cell surface markers, CD105 and CD166, is considered to be characteristic of MSCs. These are cell surface receptors involved in various biological functions, and predictive markers for the differentiation potential of MSCs [14]. In this study, these markers were identified in ADSCs along with the embryonic stem cell pluripotent markers Nucleostemin and Nanog (Figure 1(a–e)) through ICC. Nucleostemin is the stromal proliferation marker expressed in stem/progenitor cells. Meanwhile, the expression of the homeobox protein Nanog is maintaining its pluripotency. Thus, expression of these proteins indicates that the isolated ADSCs are mesenchymal stem cells with good proliferative ability and differentiation potential.

### 3.2. Intrinsic ability of IFP-derived ADSCs to become neural-lineage cells

The first essential step in any differentiation protocol is ensuring the recipient cells are permissible for the given signals and have the inherent ability to move towards the desired lineage. Kamiya et al. observed that the transcription factor ZNF521 is very efficient in driving embryonic stem cells to neural progenitors [15]; the study revealed that ZNF521 activates early neural genes epigenetically. In the current study, IFP-derived ADSCs exhibits sustained expression of ZNF521 through passage six, and thus possess the intrinsic ability to activate early neural genes (Figure 1(f)).

### 3.3. Morphological characterization using phase contrast microscopy

**Figure 2.**
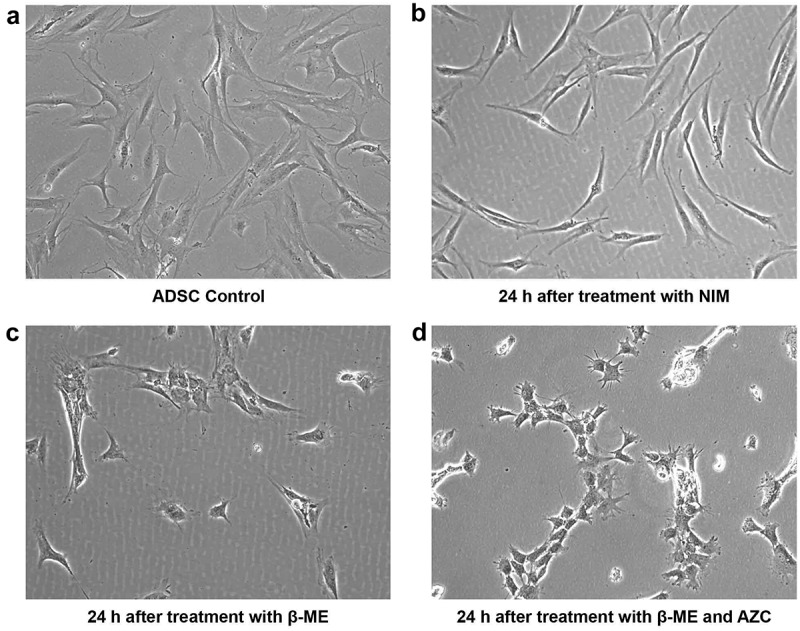
Morphological characterization of ADSCs and ADSCs after pre-induction. ADSCs expressed classic spindle-shaped fibroblastic structure of mesenchymal stem cells (a) ADSCs at 24 h after induction with only NIM exhibit sharply curved structures (b) ADSCs at 24 h after pre-induction with β-ME display cell shrinkage and clumping (c) ADSCs at 24 h after pre-induction with β-ME and 5-azacytidine (AZC) display more shrinkage, clumping, and micro-projections (d). (Magnification 10 x).

When maintained in culture medium (passage three), ADSCs appeared flat and elongated, and at d 14 exhibited spindle-shaped fibroblast morphology, forming a monolayer and adhering to the plastic surface (Figure 2(a)). Twenty-four hours after induction, in all induced groups, ADSCs lost their characteristic spindle-shaped fibroblastic morphology (Figure 2(b–d)). The cells subjected to preinduction displayed cell shrinkage. Preinduction with both 5-azacytidine and β-ME caused further shrinkage, the cells becoming very short (Figure 2(c,d)).

**Figure 3.**
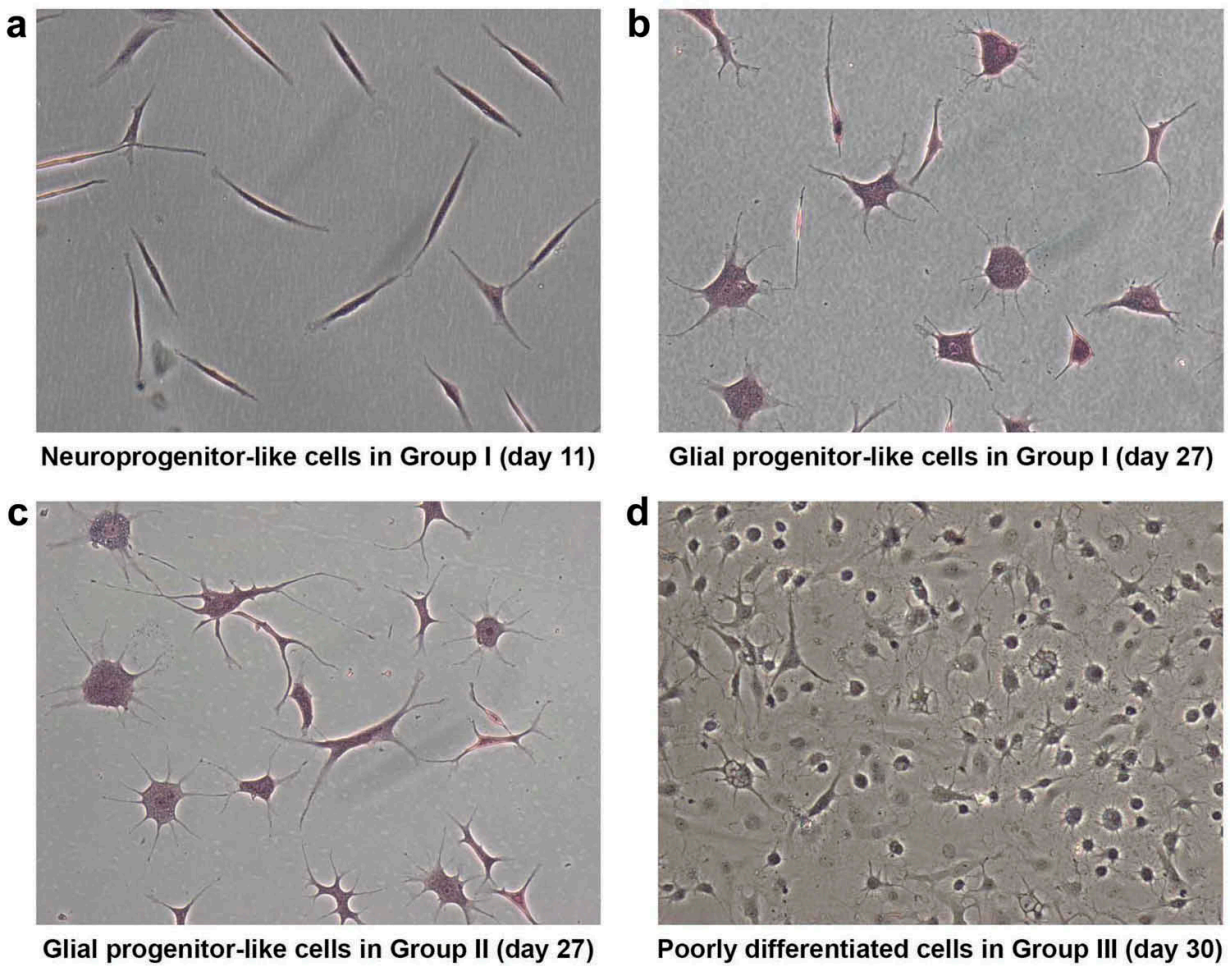
Morphological analysis using HE staining illustrates the stage-specific differentiation of ADSCs. At d 11 after induction, ADSCs treated with β-ME and NIM revealed a specific neuroprogenitor-like stage (group I) (a). At d 27 after induction, the neuroprogenitor-like cells become glial-lineage-like cells (group I) (b). At 27 d after induction, the ADSCs treated only with NIM also become glial-lineage-like cells (group II) (c). Poorly differentiated cells with small neurite extensions appeared at d 30 after neural induction with β-ME, NIM, and 5-azacytidine (group III). (Magnification10x).

At d 11, ADSCs treated with the modified Woodbury’s method [7,8] with pre-induction displayed neuronal morphology with short projections and neurite-like extensions. These are hereafter referred to as neuronal progenitor cells (NPCs) due to their characteristic resemblance to neural cells (Figure 3(a)). Upon continuous maintenance in NIM, the short neurite-like extensions of individual NPCs became well established at d 15. These NPCs then resulted in glial progenitor-like cells at d 27 after induction (Figure 3(b)). Similarly, culturing cells without pre-induction medium resulted in the formation of glial progenitor-like cells at d 27 after induction (Figure 3(c)). However, the morphologically distinct NPCs that were observed at d 11 in cells treated with pre-induction medium (Group I) were not observed at significant numbers in other groups, which stresses the importance of pre-induction.

**Figure 4.**
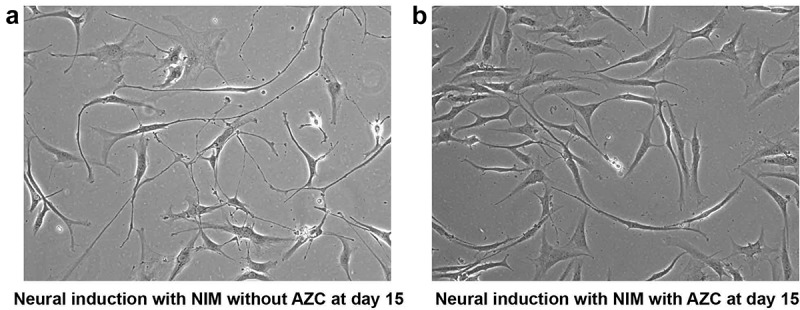
Morphological analysis of ADSCs after neural induction with and without 5-azacytidine (AZC) showed differences in differentiation pattern. At d 15 after neural induction with NIM without AZC, differentiating cells display networks resembling neural circuits (a). Non-differentiating cells at d 15 after neural induction with NIM and AZC display less differentiation (b). (Magnification10x).

Upon continuous exposure to pre-induction, NIM with 5-azacytidine for 30 d, cells did not display any significant morphological changes towards neural maturation. Instead, they remained as small round structures with minute neurite projections suggesting poorly differentiated structures (Figure 3(d)). In fact, these poorly differentiated structures were also seen much earlier, at d 15 after induction. In contrast, the control group showed long neurite extensions. This suggests a possible role for DNA methyltransferase in neural maturation (Figure 4(a,b)). Taken together, morphological analysis substantiates that NIM channelizes ADSCs towards neural-lineage cells in a stage-wise manner.

#### 3.3.1. Immunocytochemical expression and localization of specific markers confer the developmental stage, NPCs during the differentiation process

**Figure 5.**
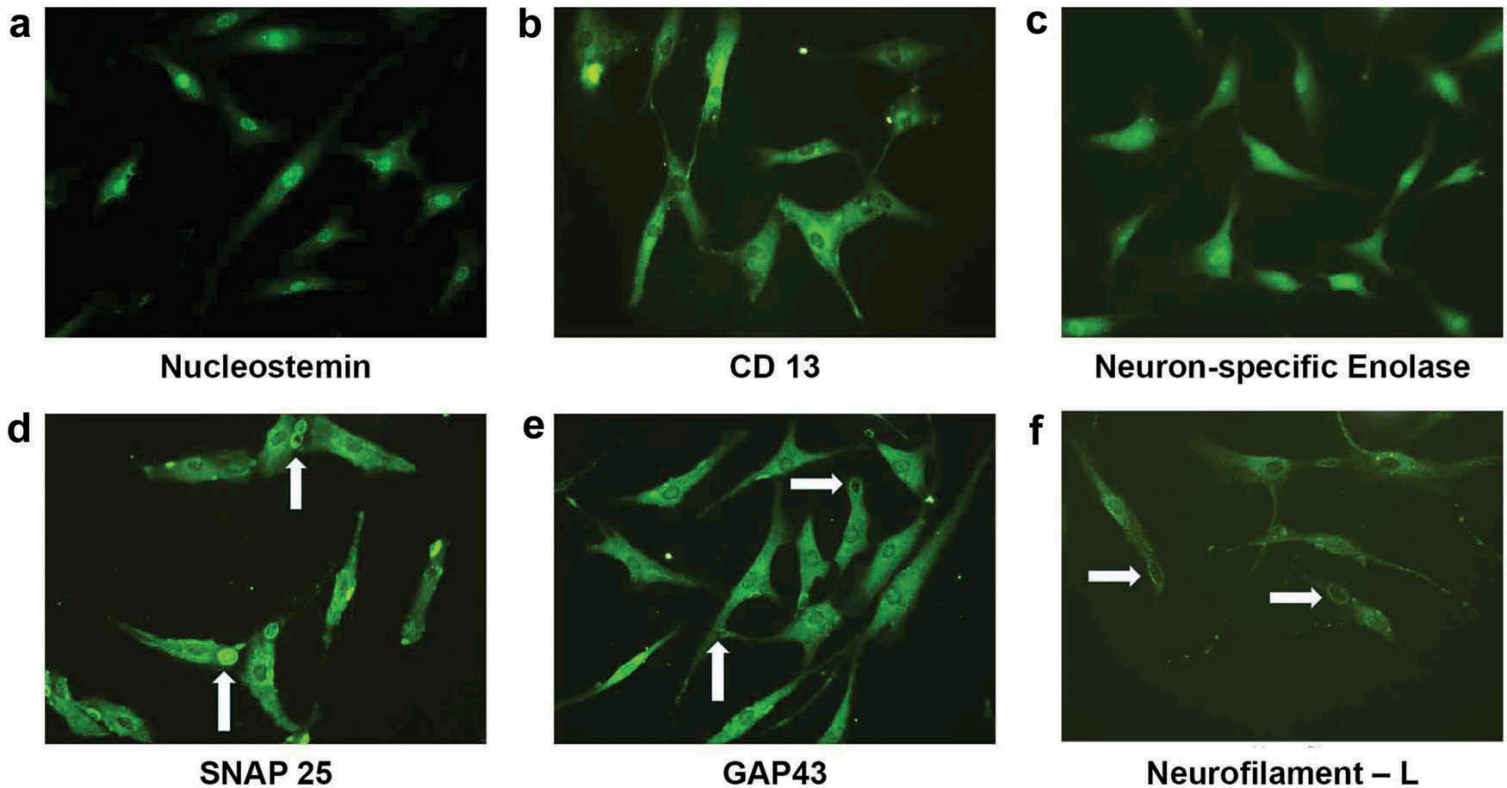
Immunocytochemical analysis on d 11 after neural induction revealed a neuroprogenitor-like stage. The proliferative marker Nucleostemin is present in the nuclei of NPCs (a). Aminopeptidase N/CD13 is concentrated around the nuclei of NPCs (b). The pan-neural protein Gamma enolase/Neuron-specific enolase is widely expressed in NPCs (c). The T-SNARE protein SNAP25, which promotes membrane fusion, is present in the growth cone and vesicles in NPCs (white arrow denotes membrane fusion) (d). Neuromodulin/GAP43 is present in the growing tips (white arrow) of the elongated axons of NPCs (e). The intermediate filament NF-L is concentrated around the nuclei and is also present in the growth cones (solid white arrow) of NPCs (f). (magnification20x). Figure 6. Semi-quantitative gene expression profiling by RT-PCR of ADSC-derived neural-lineage cells for three neural-induced samples from patients S1-3 at d 25 after induction with two different treatments: NIM with β-ME (WBME) and without β-ME (W/O BME). Untreated ADSCs used as control.

Immunocytochemistry confirmed the localization of neuronal-specific proteins at anatomical sites and thereby validated the neuroprogenitor-like characteristics of Group I cells at d 11 after induction. Specifically, these cells expressed the stem/progenitor markers NS and Aminopeptidase N, the type II transmembrane glycoprotein, CD13, NSE, GAP43, the vesicle docking and fusion protein SNAP25, and the NF-L (Figure 5). Three neural-specific proteins, GAP43, SNAP25, and NF-L, were found to localize to the growth cone of NPCs, consistent with their specific functions. The presence of SNAP25 inside the vesicles in the growth cone substantiates that the target-soluble N-ethylmaleimide-sensitive factor attachment protein receptors (t-SNARE)-mediated membrane fusion system is involved in vesicle fusion with the surface plasma membrane, which leads to neurite elongation (Figure 5(d)). GAP43 in the growth cone is necessary for the functional polarity of neuronal cells (Figure 5(e)), while NF-L is expressed earlier in neural development (Figure 5(f)) and is needed for the assembly of NF-M and NF-H, which are involved in neurite extension and are also present in the growth cone. To further confirm they are NPCs the cells were exposed to an additional signal, alternative treatment (Group IV) other than NIM (Group I).

**Figure 6.**
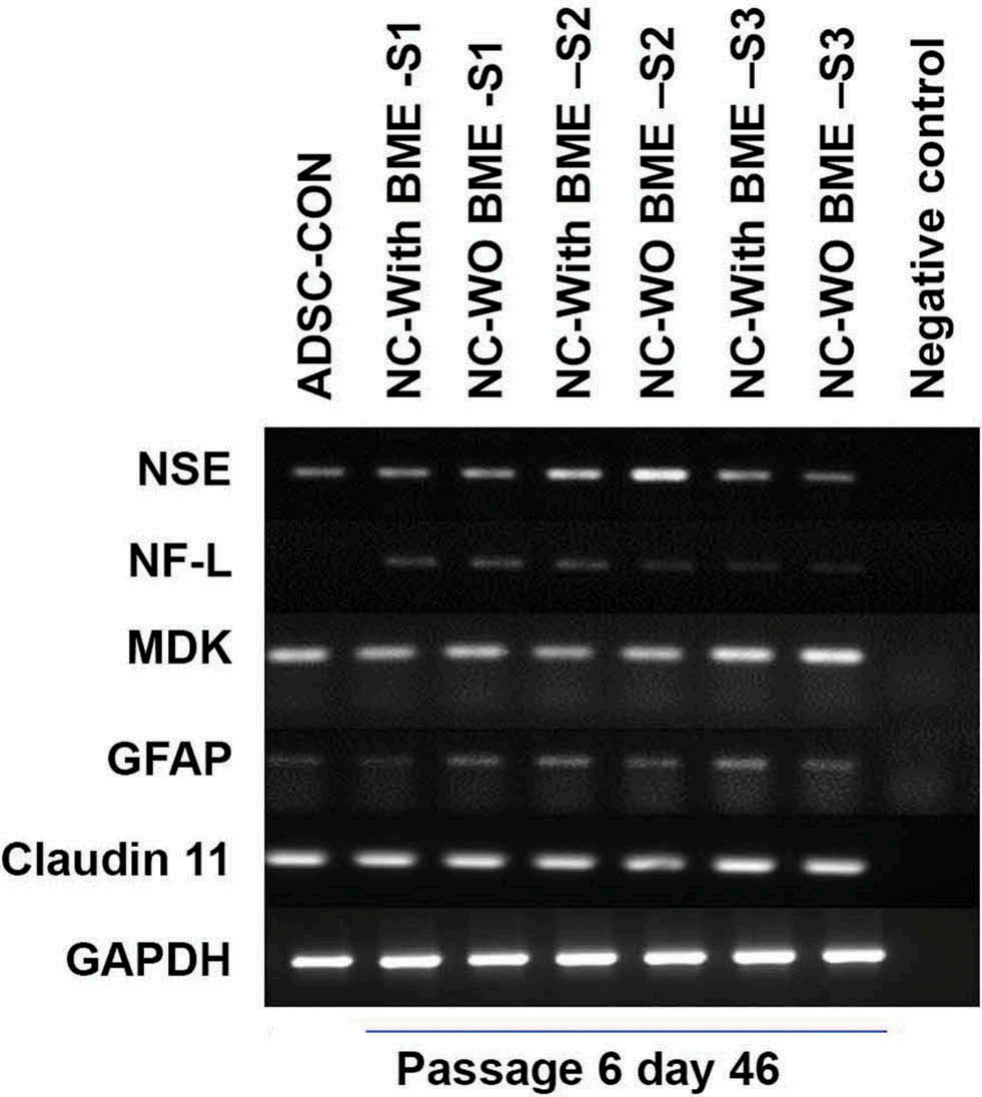
Semi-quantitative gene expression profiling by RT-PCR of ADSC-derived neural-lineage cells for three neural-induced samples from patients S1-3 at d 25 after induction with two different treatments: NIM with β-ME (WBME) and without β-ME (W/O BME). Untreated ADSCs used as control.

#### 3.3.2. Irrespective of the presence/absence of β-ME, both group I and group II cells attain character of glial-progenitor cells

Levels of specific lineage-related mRNA in Group I and Group II was analysed using semi-quantitative RT-PCR analysis. There was no significant difference in the expression of mRNA levels in between groups. Gene expression analysis validated that the resultant cells were glial-progenitor cells; since after 25 d of after induction, both the induced groups faintly expressed NF-L and Glial fibrillary acidic protein (GFAP). The positive expression of claudin 11 (a major four-transmembrane protein classified under the claudin family of tight junction proteins and also known as Oligodendrocyte specific protein [OSP]), and midkine (a heparin-binding neurite promoting factor) at d 25 after induction at the messenger RNA level further endorses the transdifferentiation of ADSCs towards neural lineage. GAPDH Expression of Glyceraldehyde-3-Phosphate phosphate dehydrogenase (GAPDH) expression was used as an endogenous control. RT-PCR results are shown in Figure 6.

**Figure 7.**
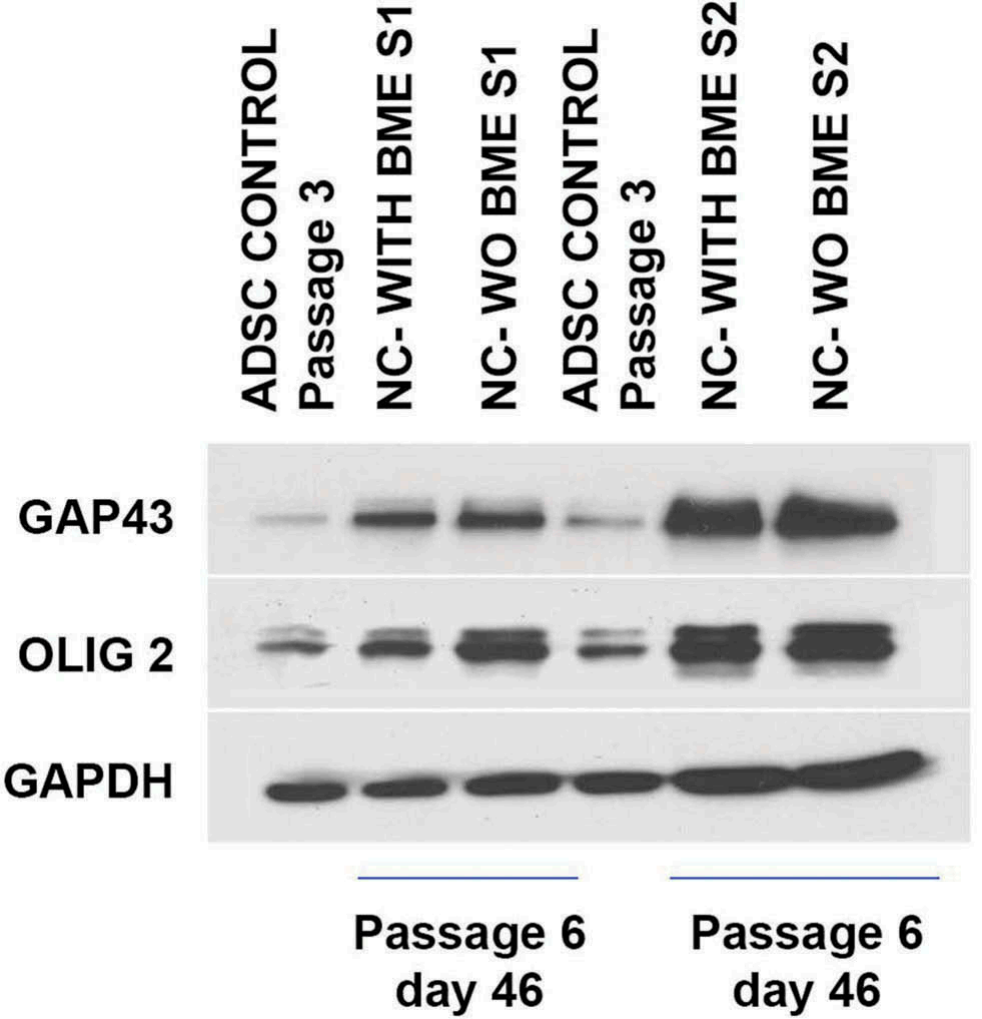
Western blot analysis of ADSC-derived neural-lineage cells for two neural-induced samples from patients S1 and S2 at d 25 after induction with two different treatments: NIM with β-ME (WBME) and without β-ME (W/O BME). Untreated ADSCs used as control.

Further, the expression of proteins associated with glial lineage was determined by western blot. Control ADSCs expressed very negligible amounts of GAP43 and OLIG2 in comparison to the induced groups. The extensive expression of these proteins in induced groups at d 25 after induction (Figure 7) supports that they are the glial-progenitor cells. GAPDH expression was used as an endogenous control.

#### 3.3.3. Treatment with an inhibitor of epigenetic modulation results in poorly differentiated structures with short neurite projections

**Figure 8.**
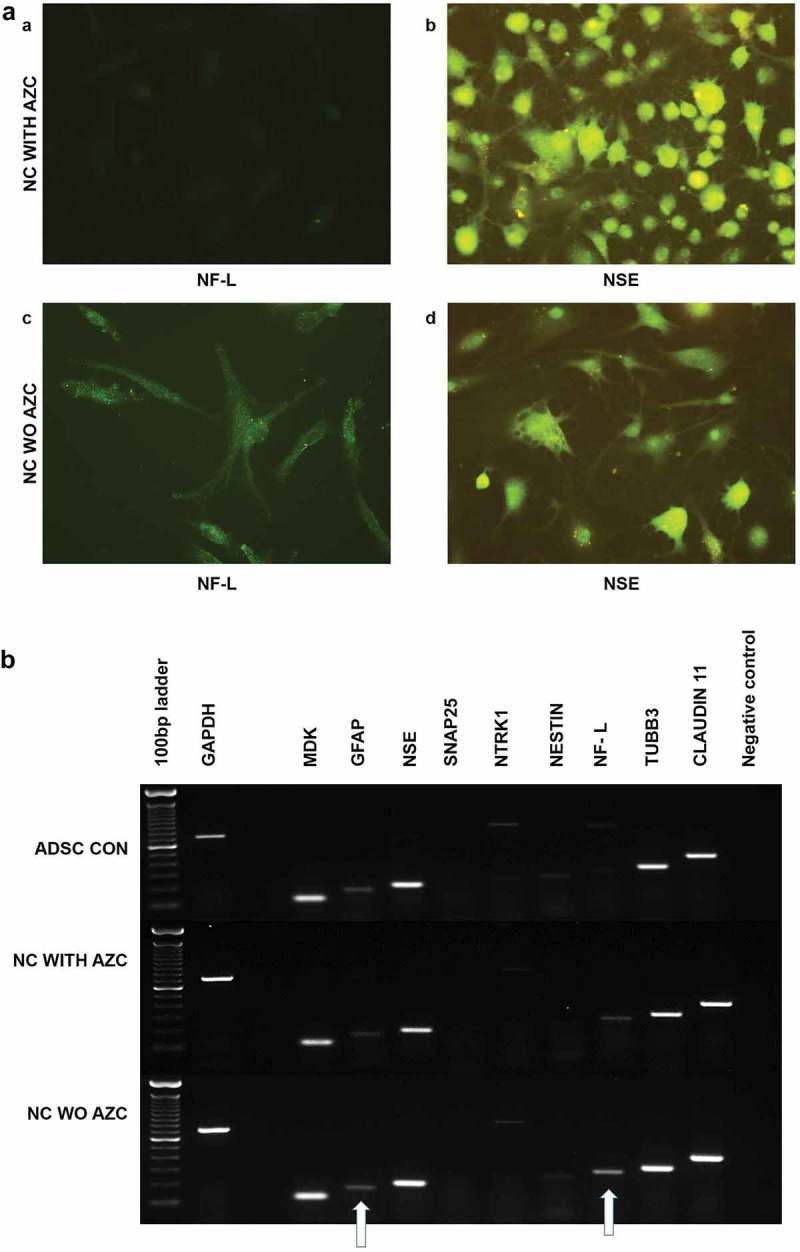
Immunocytochemical analysis of ADSCs on d 30 after induction (a), with β-ME, NIM, and AZC showed NSE positivity and NF-L negative. The resultant cells are spheroids with minute neurite projections, resembling poorly differentiated structures (a, b). ADSCs on d 30 after induction with β-ME and NIM without AZC showed NSE and NF-L positivity. They resultant cells are not spheroids (c, d) (Magnification 20 x). Semi-quantitative gene expression profiling by RT-PCR of ADSC-derived neural-lineage cells at d 30 after induction (b). Neural – lineage cells obtained through the treatment of NIM without 5-azacytidine (NC WO AZC) and Neural – lineage cells obtained through the treatment of NIM with 5-azacytidine (NC with AZC). Untreated ADSCs used as control. The analysis shows reduced expression of neurofilaments.

Visualization of the neuron-specific glycolytic protein NSE through ICC revealed that ADSCs treated with 5-azacytidine were NSE-positive with immature neural morphology at d 30 after induction (Figure 8(a)). They were also negative for NF-L. The treatment may have resulted in retarded radial axonal growth, in which NF-L and NF-M play crucial roles. However, the ADSCs treated without 5-azacytidine exhibits NSE positivity with different morphology, at the same time the exhibit mild positivity for NF-L (Figure 8(b)).

Semi-quantitative gene expression analysis of intermediate filaments comparing the cells treated with and without 5-azacytidine revealed that the DNA methyltransferase inhibitor suppresses the expression of Neurofilaments. Thus, DNA methyltransferase appears to be important for neuronal differentiation (Figure 8(c)).

#### 3.3.4. Accomplishment of complete neuronal differentiation by an alternative treatment other than NIM confers that NPCs could result in two variable outcomes depending on the signals provided

**Figure 9.**
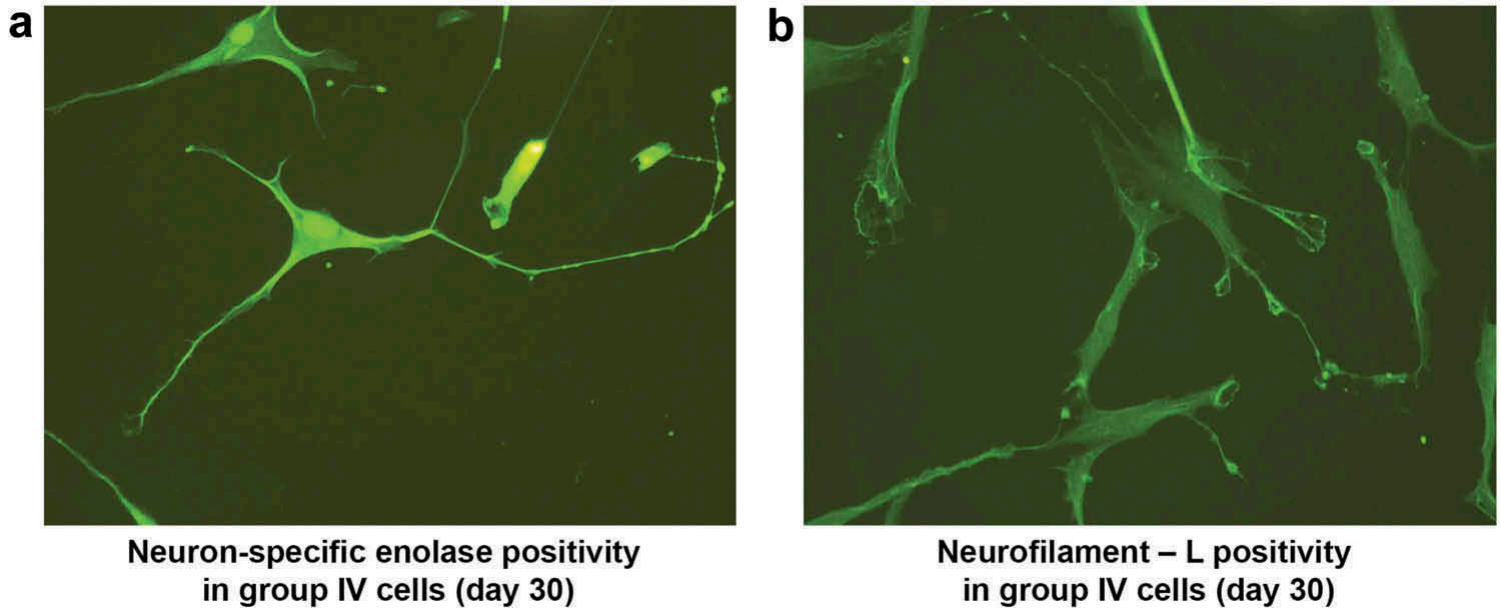
Immunocytochemical analysis of neuroprogenitor-like cells on d 30 after alternative treatment with bFGF and antioxidants showed NSE positivity in neuron-like cells. Cells exhibited a pyramidal neuron-like structure with a cone-shaped cell body (a). NF-L positivity in the filaments of neuron-like cells. NF-L was also present in growth cones, unlike progenitor cells; the filaments are long denoted by the white arrow (b). (Magnification20x).

After the alternative treatment with 50 ng of bFGF and BHA, NPCs continued to differentiate until d 30 after induction. The resultant cells possessed a cell body, axon, and dendritic structures resembling a typical neuron, and were immunocytochemically positive for NSE and NF-L. (Figure 9(a,b)). ADSCs maintained in culture medium without induction showed negative results for all the neural markers examined.

**Figure 10.**
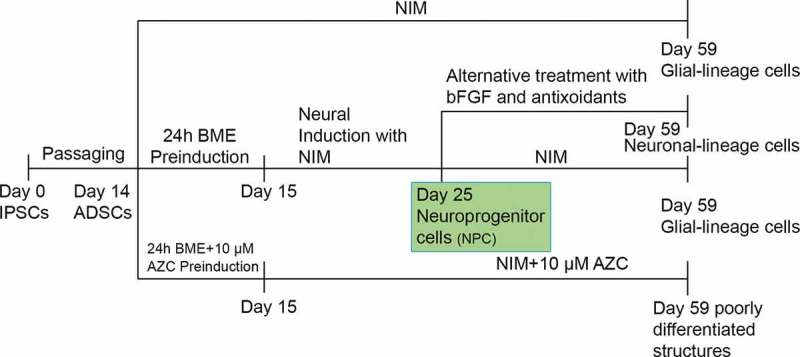
Schematic representation of protocols and the results were depicted.

A schematic representation of the protocols and results is depicted in Figure10.

## 4. Discussion

The experiments performed in this study demonstrate the transdifferentiation of infrapatellar fat pad-derived ADSCs to neural-lineage cells by the modified Woodbury’s method [7,8]. From these results, we conclude that the inherent ability of ADSCs enables them to upregulate neural lineage-specific genes in a temporal manner with minimal culture conditions. The gene expression profiles of these stage-specific neural-lineage cells derived from ADSCs are similar to those of the corresponding stages of neural maturation.

Three sequel studies [16–18] have re-evaluated the chemical induction protocol originally demonstrated by Woodbury [7,8] and reported that the appearance of neurite-like structures in ADSCs within a few hours of induction was due to actin depolymerization and cytoskeletal disruption. Furthermore, they also stated that the observed increase in neuron-specific immunostaining positivity was due to an accumulation of protein resulting from the aforesaid actin depolymerization and shrinkage of cells [16–18]. Unlike Woodbury [7,8] and its sequel works [16–18], in which cells displayed neuronal markers hours after exposure to β-ME, the transdifferentiation of ADSCs in this study was observed over an extended period of 45 d after exposure to NIM and β-ME, with progressive changes in gene expression. Although ADSCs exposed to β-ME tended to shrink in the first 24 h, previously reported as an ‘artefact’, this phenotype was found to revert back to a fibroblast-like structure once NIM was added.

At d 11 after treatment, with both pre-induction and NIM (Group I), ADSCs transformed into morphologically distinct, transit-amplifying cells called neuroprogenitor cells (NPCs) that are not distinguishable in either the group without pre-induction or the group with 5-azacytidine. Though β-ME and BHA are strong antioxidants, they have been shown to enhance the survival of neurons [19], this study reveals the importance of pre-induction with β-ME to attain the NPC stage.

In this study, the identified NPCs are supported not only by the mere expression of a new array of progenitor-specific markers but also by their expression at specific localizations within the cell. The cells at this stage possess good mitotic capacity and the ability to differentiate into both glial and neuronal subtypes, responding to provided growth signals. Furthermore, Nucleostemin, an evolutionarily conserved protein that endows a controlled proliferation rate to stem/progenitor cells of neuronal origin [20] and to ES-derived neural stem/progenitor cells [21] has been found to be present in the nucleus of NPCs. In addition, Aminopeptidase N (CD13), which is believed to be involved in the inactivation of neural peptides present in both neuronal- and glial-lineage cells, is also expressed in NPCs. Three other neural-specific proteins, Neuromodulin (GAP43), SNAP25, and NF-L were localized to the growth cones of NPCs. Localization of GAP43 to the growth cone confirms differentiation towards neuronal lineage [22]. GAP43 is considered a ‘pioneer’ of neural differentiation as it has been found to be expressed in multipotent ectodermal precursors prior to neuro-ectodermal differentiation [23–25]. The localization of SNAP25 to vesicles in the growth cone of NPCs substantiates that the t-SNARE-mediated membrane fusion system is involved in the process of vesicle fusion with the surface plasma membrane that leads to neurite elongation [26,27]. At later stages, the expression of SNAP25 was found to be totally lost, consistent with the lack of this protein in glial cells. Finally, NF-L, which was expressed in both the growth cone and perikaryon of NPCs (Figure 5), is considered to be an early neural marker involved in the induction of neural differentiation [28]; however, there is no previous report on its pattern of expression at different progenitor stages.

In general, the CNS is repopulated with NPCs that differentiate into neuronal/glial cells according to necessity indicated by signals from damaged neural tissue; these cells also aid in long-term neural integration, remodelling, and regeneration [29]. The myriad of phenotypic and genotypic variation in differentiated neural tissue is produced through the different modes of symmetric and asymmetric division that occur in NPCs. These divisions permit them to differentiate into neuronal or glial progeny [30]. Hence, the success of neural tissue replacement is purely dependent on the potential of ‘determined NPCs’. Therefore, to evaluate whether the NPCs identified in our study mimic those cells that repopulate the CNS, NPCs were first exposed to NIM and later subjected to alternative treatment with 50 ng/ml of bFGF and BHA. Namely, bFGF is an established neurogenic factor in neural induction that has been extensively studied [31,32], and alternating bFGF and BHA mimics the temporal exposure of bFGF during development that signals progenitor cells to become neuronal-lineage cells [33].

Our results confirm that continuous exposure of NPCs to NIM promoted their differentiation towards glial lineage, as evidenced by the sequential expression across 45 d of culture of glial-specific genes like OLIG2, a helix-loop-helix transcription factor; midkine, a neurite growth-promoting factor; and claudin 11, a component of the integral membrane and tight junction. These proteins together play pivotal roles in orchestrating oligodendrocyte development [34–39]. Furthermore, the continually increasing expression of *NSE* during the process of maturation suggests that the resultant cells are oligodendrocyte precursors and not mature cells [40]. Expression of *NSE* may aid in the development of large quantities of membrane structures and protoplasmic processes [41]. Finally, the presence of GAP43 in glial precursors and oligodendrocytes from our study confirms its previously reported role in CNS development [40]. Thus, we have identified NPCs in a long-term culture condition whose differentiation results in variable outcomes depending on the signals given. Further studies are needed to analyse these NPCs, particularly in terms of terminal differentiation and the underlying molecular mechanisms, which need to be understood in order to realize the complete potential of NPCs for cell therapy.

We additionally confirmed in this study that the transdifferentiation of ADSCs to neural lineage is influenced by epigenetic factors. 5-Azacytidine treatment significantly reduces the global level of DNA methylation in adipose tissue – derived mesenchymal stem cells [42,43]. The blocking of DNA methyltransferase enzymes (DNMT1, DNMT3a, and DNMT3b) by 5-azacytidine at early stage interrupted both progenitor cell differentiation and neuronal/glial maturation, resulting in immature neural cells with NSE positivity. Moreover, semi-quantitative gene expression analysis of cells treated with and without 5-azacytidine revealed that 5-azacytidine suppresses the expression of neurofilaments, which is an indispensable structural element of axons [44]. Although refinement of the current study is essential to show the inhibition of DNMT, these findings are consistent with previous reports on the role of DNA methyltransferases in CNS maturation and function during development [45–47].

In conclusion, irrespective of the presence of β-ME, the modified Woodbury’s chemical induction protocol [7,8] resulted in glial progenitors. On subsequent exposure to NIM induced ADSCs sequentially acquired markers associated with NPCs and later glial progenitor cells. In addition, giving two different signals to the NPCs resulted in distinct outcomes, glial progenitors and neuronal cells. The overall differentiation process is controlled by the epigenetic modulator DNA methyltransferase (DNMT), as an amended protocol using 5-azacytidine resulted in immature neural-lineage cells with short projections. Further study of the occurrence and terminal differentiation of NPCs is needed to support them as a promising source for neural tissue replacement.
